# Uremic Toxins and Hemodiafiltration: From Molecular Mechanisms to Clinical Outcomes

**DOI:** 10.3390/toxins18070312

**Published:** 2026-07-17

**Authors:** Stefano Stuard, Charles Hugh-Jones, Dinesh Chatoth, Michael Anger, Alfred Gagel, Bernard Canaud

**Affiliations:** 1 Global Medical Office, Fresenius Medical Care, 26020 Palazzo Pignano, Italy; 2 Global Medical Office, Fresenius Medical Care, Waltham, MA 02451, USA; charles.hugh-jones@freseniusmedicalcare.com (C.H.-J.); dinesh.chatoth@freseniusmedicalcare.com (D.C.); michael.anger@freseniusmedicalcare.com (M.A.); 3 Global Research and Development, Fresenius Medical Care, 61352 Bad Homburg, Germany; alfred.gagel@freseniusmedicalcare.com; 4 School of Medicine, Montpellier University, 9 Rue des Carmelites, 34090 Montpellier, France; canaudbernard@gmail.com

**Keywords:** uremic toxins, hemodiafiltration, middle molecules, protein-bound uremic toxins, β2-microglobulin, end-stage kidney disease

## Abstract

Uremic syndrome results from the accumulation of biologically active solutes that contribute to inflammation, oxidative stress, endothelial dysfunction, cardiovascular disease, anemia, CKD–mineral and bone disorder, and protein-energy wasting in patients with end-stage kidney disease. While conventional hemodialysis efficiently removes small water-soluble compounds, clearance of middle molecules and protein-bound uremic toxins remains limited. Post-dilution hemodiafiltration (HDF) combines diffusion and convection to enhance removal across a broader molecular-weight spectrum. This review summarizes the mechanisms of uremic toxin accumulation, the biologic and clinical relevance of major retained solutes, and current evidence supporting improved toxin removal with HDF. Randomized trials, meta-analyses, and observational studies have associated HDF with reduced inflammatory burden, improved hemodynamic tolerance, and favorable clinical outcomes, supporting its role as an advanced strategy for targeting the broader spectrum of uremic toxicity.

## 1. Introduction

Uremic syndrome is a systemic disorder resulting from the disruption of the internal milieu’s homeostasis and the accumulation of biologically active solutes, electrolytes, and water that are normally excreted or metabolized by healthy kidneys, leading to clinical symptoms. In addition, the loss of renal endocrine (e.g., erythropoietin secretion, vitamin D activation) and metabolic functions (e.g., protein catabolism) further contributes to the development of the syndrome. Progressive kidney failure leads to the accumulation of organic and inorganic compounds in plasma and tissues, and to endocrine dysfunctions that contribute to a wide range of cardiovascular, inflammatory, metabolic, neurologic, endocrine, and immunologic abnormalities. Uremic toxicity, therefore, represents far more than a simple biochemical disturbance; it is a complex, multisystem disorder driven by the accumulation of retained solutes and the modification of others, which together disrupt multiple pathophysiologic pathways, including cellular signaling and receptor-mediated processes, as well as interorgan communication. Historically, urea was the first recognized uremic retention solute, and dialysis adequacy has long been assessed primarily through urea kinetic parameters. However, advances in metabolomics, proteomics, and molecular biology have demonstrated that the uremic milieu is substantially more complex than can be explained by small-solute kinetics alone. The native kidney regulates solute homeostasis through glomerular filtration, tubular secretion, tubular reabsorption, intracellular metabolism, and continuous endocrine and metabolic regulation. Conventional intermittent hemodialysis reproduces only a portion of the kidney’s excretory function and predominantly removes small water-soluble compounds through diffusion.

The European Uremic Toxins (EUTox) Working Group of the European Society for Artificial Organs proposed a clinically relevant classification of uremic retention solutes according to their physicochemical properties, which largely determine extracorporeal removal efficiency [1]. The group has also developed a publicly accessible database that provides regularly updated information on the classification, physicochemical characteristics, biological effects, and reported concentrations of uremic solutes [2]. More recently, metabolomics-based approaches have expanded this framework by identifying additional retained solutes, emphasizing that the uremic milieu is more complex than previously recognized and that not all retained compounds necessarily exert harmful biological effects [3]. Recent reviews have also emphasized the need to integrate physicochemical classification with biological activity, clinical relevance, and advances in blood purification technologies to better understand the contribution of uremic solutes to CKD complications [4].

Uremic toxins are broadly categorized into small water-soluble compounds, middle molecules, and protein-bound uremic toxins (PBUTs) [5,6]. Small water-soluble compounds, including urea and creatinine, are efficiently removed by conventional hemodialysis through diffusion. In contrast, middle molecules, generally ranging from approximately 0.5 to 40–45 kDa, are less efficiently removed by diffusion alone and benefit from convective transport. PBUTs, including indoxyl sulfate and p-cresyl sulfate, exhibit strong albumin binding, substantially limiting extracorporeal removal because only the free circulating fraction can cross the dialysis membrane [7]. As a result, several biologically active uremic solutes implicated in inflammation, cardiovascular injury, oxidative stress, endothelial dysfunction, and immune dysregulation may persist despite apparently adequate clearance of small-solutes assessed by conventional urea-based metrics.

Recognition that conventional hemodialysis (HD) inadequately removes larger uremic solutes has driven the development of convection-based therapies. Online hemodiafiltration (HDF) has been consistently associated with improved clinical outcomes compared with conventional HD, particularly when post-dilution convection volumes exceed 23 L per session (high-volume HDF, HVHDF) [8,9,10,11,12,13,14,15,16,17,18,19,20,21,22,23]. HVHDF combines efficient diffusive clearance of small solutes with enhanced convective removal of middle molecules and larger uremic solutes. Its superior solute removal has been confirmed using global removal score approaches that incorporate solutes ranging from 60 Da to 41 kDa while accounting for albumin loss [24,25,26]. These observations are supported by a recent systematic review and meta-analysis of 24 randomized controlled trials including 6072 patients, which showed greater removal of β2-microglobulin and other uremic toxins, lower inflammatory marker concentrations, reduced erythropoiesis-stimulating agent requirements, and improved phosphate control with HDF than with HD [27]. Table 1 summarizes uremic toxins for which increased clearance with HDF vs. high-flux HD has been demonstrated, along with their clinical relevance. In addition to dialysis modality, overall solute removal is strongly influenced by treatment schedule, particularly total weekly treatment time, with longer treatment durations associated with improved toxin clearance. Residual kidney function also plays a crucial role, substantially contributing to the removal of larger-molecular-weight (MW) solutes and protein-bound uremic toxins that are less efficiently cleared by extracorporeal therapies.

## 2. Mechanisms of Uremic Toxin Accumulation

Uremic toxin accumulation in chronic kidney disease (CKD) and end-stage kidney disease (ESKD) reflects the interplay of multiple pathophysiologic mechanisms that extend beyond reduced glomerular filtration rate (GFR). These include impaired renal clearance, loss of tubular secretory function, intestinal dysbiosis, oxidative stress, chronic inflammation, altered protein binding, tissue redistribution, and the inability of dialysis therapies to reproduce native kidney function [1,76,77,78]. The healthy kidney continuously maintains solute homeostasis through glomerular filtration, tubular secretion, tubular reabsorption, and intracellular metabolism. Conventional dialysis replaces only a fraction of these excretory functions, primarily by diffusively removing small, water-soluble compounds. Therefore, many biologically active compounds with middle and large MW accumulate, contributing to a residual uremic syndrome despite apparently adequate dialysis delivery, as assessed by conventional metrics such as Kt/V [1,77,78]. These observations highlight the need not only to enhance the removal of middle and larger molecules through therapies such as HDF, but also to develop complementary adequacy metrics that better reflect the clearance of these clinically relevant solutes.

Residual kidney function remains clinically relevant even at low levels, as residual tubular secretion and metabolic activity contribute substantially to the clearance and control of uremic toxins, particularly middle molecules and PBUTs [79,80]. Reduced GFR remains the principal determinant of retention of small water-soluble compounds, including urea, creatinine, uric acid, and guanidino compounds [1,81]. Some compounds accumulate proportionally with declining kidney function, whereas others increase disproportionately due to changes in tubular transport, metabolic generation, or extrarenal clearance pathways. Loss of proximal tubular secretory function is particularly important for the accumulation of PBUTs. Under physiologic conditions, many protein-bound solutes are actively secreted through organic anion transporters, including OAT1 and OAT3 [82,83,84]. As tubular transport progressively declines, plasma concentrations of indoxyl sulfate, p-cresyl sulfate, indole acetic acid, and other PBUTs increase substantially. Because these compounds are highly albumin-bound, conventional dialysis removes only the free circulating fraction, limiting extracorporeal clearance [82,83,85].

The gut contributes significantly to the generation of uremic toxins. CKD alters gut microbiota composition and intestinal barrier integrity, favoring increased production and systemic absorption of toxic metabolites [86,87,88]. These mechanisms amplify systemic inflammation and oxidative stress, contributing to endothelial dysfunction and cardiovascular injury.

Oxidative stress and chronic inflammation are hallmarks of the uremic state. Retained uremic solutes promote inflammatory activation, while inflammation itself further increases toxin generation and biologic toxicity, creating a self-perpetuating cycle [77,89,90,91,92]. Protein binding further complicates toxin kinetics and limits their removal. In chronic kidney disease, particularly in patients with diabetes, both the concentration and functional integrity of albumin are impaired. This yields a higher free fraction of many clinically relevant uremic toxins, which are normally >90% albumin-bound [82,85] and results in aggravated exposure of body tissues and the endothelium to these chemically reactive substances. Moreover, oxidative stress, carbamylation, and glycation may alter albumin structure and binding properties, potentially increasing the biologically active free fraction of these toxins.

Because extracorporeal therapies predominantly remove the unbound fraction, strong protein binding remains a major barrier to the effective clearance of many uremic toxins with current dialysis techniques.

Tissue compartmentalization further limits the effective removal of uremic toxins during highly efficient extracorporeal therapies. Many middle molecules and PBUTs are widely distributed in poorly perfused or remote tissue compartments, thereby restricting their immediate availability for extracorporeal clearance. As a result, significant post-dialytic rebound occurs as these solutes redistribute from peripheral tissues back into the plasma compartment following treatment [78,93]. The compartmental kinetics of many middle molecules, larger solutes and PBUTs also have practical implications for dialysis prescription. Their slow transfer from remote areas (e.g., cells, tissues) to the circulating vascular compartment, as reflected by low intercompartmental mass transfer coefficients, limits intradialytic removal. Consequently, longer treatment durations and more frequent dialysis sessions may improve their overall removal by allowing more complete tissue-to-plasma equilibration during dialysis. These considerations indicate that optimizing uremic toxin removal depends not only on the dialysis modality and delivered convective volume, but also on the overall treatment schedule, including adequate weekly treatment time. However, the relative contribution of treatment time and convective volume to the removal of individual uremic toxins remains incompletely defined.

## 3. Dialysis Modalities and Uremic Toxin Removal

**Figure 1:**
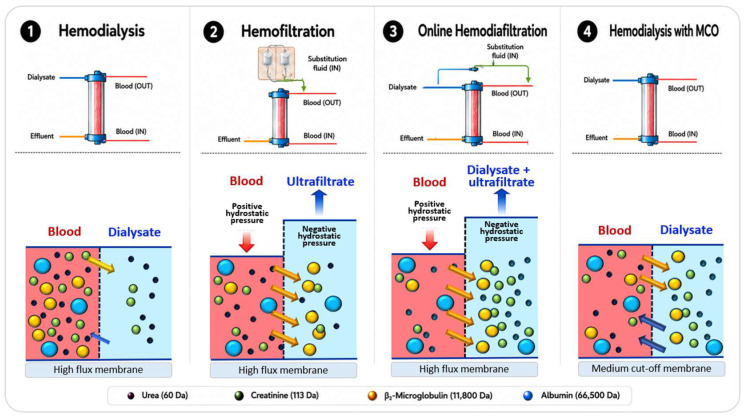
Mechanisms of Solute Removal in hemodialysis (1), hemofiltration (2), online hemodiafiltration (3), and hemodialysis with a medium cut-off dialyzer (4). Legend. Schematic representation of diffusion and convection (orange arrows), and backfiltration from dialysate into blood compartment (blue arrow) across different dialysis techniques. High-flux dialysis, hemofiltration, hemodiafiltration, and medium-cut-off dialysis are compared to illustrate the effects of membrane permeability and hydrostatic pressure gradients on the removal of small-, medium-, and large-molecular-weight solutes.

Conventional HD primarily relies on diffusive transport and efficiently removes small water-soluble solutes. However, removal efficiency progressively decreases with increasing molecular size because diffusion coefficients decline substantially for larger compounds. Based on membrane permeability characteristics, HD may be classified as low- or high-flux. High-flux membranes exhibit greater hydraulic permeability and permit higher internal filtration and convective transport in addition to diffusion. In conventional HD, however, solute removal remains predominantly diffusion-driven, with transport occurring down concentration gradients between blood and dialysate compartments. Diffusion is particularly effective for low-MW solutes such as urea and creatinine because diffusion coefficients decrease progressively with increasing MW. Consequently, conventional HD provides efficient clearance of small solutes but more limited removal of larger middle molecules, even when these molecules are theoretically permeable across the dialysis membrane (Figure 1(1)).

Hemofiltration (HF) was subsequently developed to improve the removal of middle- and large-MW solutes. By relying primarily on convective transport, HF achieves much greater clearance of middle molecules than conventional HD (Figure 1(2)). However, it is less efficient at removing small solutes due to the limited transport capacity of the available plasma water for filtration. It requires large volumes of sterile replacement fluid, thereby increasing treatment complexity, resource utilization, and cost.

HDF was developed to combine the diffusive efficiency of HD for small solutes with the convective advantages of HF for large molecules. By integrating diffusion and convection into a single treatment, HDF enhances removal across the whole MW range up to albumin (Figure 1(3)).

**Figure 2:**
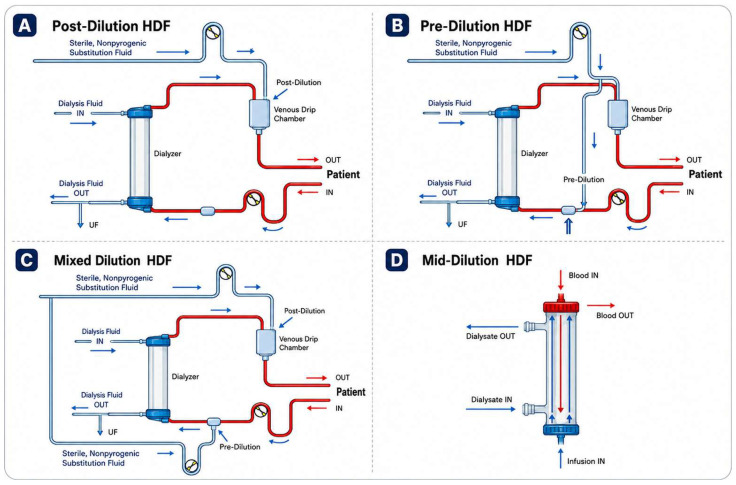
Different types of hemodiafiltration techniques. Legend. (**A**): Online post-dilution HDF; (**B**): Online pre-dilution HDF; (**C**): Online mixed-dilution HDF; (**D**): Online mid-dilution HDF.

Post-dilution online HDF is currently the most widely used configuration (Figure 2B) because it offers the highest total efficiency and especially superior removal of middle molecules, generally defined as solutes between approximately 500 Da and 45 kDa, which are incompletely cleared by conventional HD. Enhanced removal of these molecules has been associated with attenuation of inflammatory burden and improved cardiovascular risk markers. Post-dilution HDF is the preferred modality because it maximizes convective transport and overall depurative efficiency. Randomized trials [8,9,10,11,12], individual participant data meta-analyses [13,14], and large observational studies [15,16,17,18,19,20,21,22,23,94] have consistently shown that HDF is associated with a convective volume-related improved clinical and patient-reported outcomes compared with conventional HD, particularly when convection volumes >23 L/session are achieved [10,12,13,14,20,21,22,23,95]. Consequently, high-volume post-dilution HDF (HVHDF) is increasingly adopted in routine dialysis practice [96].

Successful delivery of HVHDF requires adequate vascular access and an optimized dialysis prescription, including the use of a high-flux dialyzer with an ultrafiltration coefficient of at least 50 mL/h/mmHg, a blood flow rate of at least 340 mL/min, a treatment time of 220–240 min, and HDF systems capable of safely delivering high convection volumes by continuously adjusting substitution flow during treatment. Together, these elements enable the consistent achievement of convection volumes > 23 L/session [97]. Systems that also adjust dialysate flow according to blood flow can further improve dialysate efficiency while reducing dialysate use, thereby lowering water consumption [98].

Hemodialysis using medium-cut-off (MCO) membranes was developed as a further step, enhancing the removal of larger middle-sized molecules while preserving the operational simplicity of conventional HD. MCO membranes exhibit larger and more heterogeneous pore-size distributions than conventional high-flux membranes, improving the clearance of molecules up to approximately 45 kDa [99,100,101,102].

Unlike online HDF, where convection is externally generated by dedicated substitution pumps to actively reach and maximize the prescribed convective volume, MCO membranes rely on passively internally generated filtration and backfiltration phenomena occurring along the dialyzer fibers, a process sometimes referred to as internal hemodiafiltration [102,103,104,105] (Figure 1(4)).

Experimental studies have estimated internal convective volumes of approximately 7–13 L/session, although the delivered convective dose cannot be directly measured or controlled in routine clinical practice [106,107]. The achieved convective volume is strongly influenced by hematocrit-dependent changes in blood viscosity. At low hematocrit levels, exchange volumes may remain limited despite the availability of a large plasma water fraction for filtration. Conversely, higher hematocrit levels increase blood viscosity, promote internal filtration, and enhance blood thickening within the dialyzer. Excessive hemoconcentration may impair filtration efficiency and increase the risk of membrane fouling and clotting. This phenomenon is particularly relevant during the latter stages of treatment, when ultrafiltration-induced volume depletion further elevates hematocrit and blood viscosity, especially in dialyzers designed to maximize internal filtration.

Compared with conventional high-flux HD, MCO membranes improve the removal of larger middle molecules, including free light chains and selected inflammatory mediators, but are also associated with greater albumin permeability [100,101,108,109,110,111,112,113,114,115]. The greater albumin permeability of MCO membranes is associated with modest intradialytic albumin losses during hemodialysis, as these membranes are not intended for use in HDF. The long-term clinical relevance of this albumin loss remains uncertain and warrants further investigation in adequately powered long-term studies.

Although preliminary studies suggest potential biologic and clinical benefits, robust long-term outcome data remain limited, and large randomized studies comparing MCO-based HD with online HDF are ongoing [116,117].

More frequent or longer home hemodialysis can also provide excellent uremic solute removal by increasing treatment frequency and duration. Because this review focuses on conventional in-center hemodialysis and hemodiafiltration, home hemodialysis is not discussed further, although it remains an important treatment option for selected patients.

## 4. Quantification of Solute Removal in Hemodiafiltration

HDF combines diffusive and convective transport mechanisms to enhance the removal of uremic solutes across a broad MW spectrum. Unlike conventional HD, where diffusion is the predominant mechanism, HDF incorporates substantial convection generated by ultrafiltration and the infusion of substitution fluid. Consequently, solute clearance during HDF depends on several interacting factors, including blood flow rate and blood properties, dialysate flow rate, convection volume, the membrane’s sieving characteristics, dilution mode, and the solute’s physicochemical properties. Instantaneous solute clearance during HDF can be assessed using two complementary approaches.

The first approach, commonly used in clinical practice, is based on direct measurements from blood or dialysate samples collected during treatment. Blood samples are collected from the arterial and venous blood lines after complete mixing of blood and substitution fluid (Table 2, Equation (2)). Alternatively, dialysate-side clearance may be determined more accurately from dialysate measurements (Table 2, Equation (3)). Both methods yield identical results within measurement uncertainties and provide a direct quantification of extracorporeal solute removal under clinical operating conditions.

The second approach is theoretical and estimates solute clearance based on the dialyzer’s intrinsic characteristics, such as the mass-transfer area coefficient and the sieving coefficient, which are specific for each solute. These parameters are combined with treatment operating conditions, including blood flow, dialysate flow, substitution flow, and the dilution factor (DF) associated with the selected HDF mode. The total instantaneous extracorporeal clearance during HDF may therefore be approximated as the sum of diffusive and convective clearances, adjusted for the dilution factor according to the mode of replacement fluid infusion (Table 2, Equations (4) and (5)) [118].

## 5. Clinical Consequences of Uremic Toxin Accumulation

**Figure 3:**
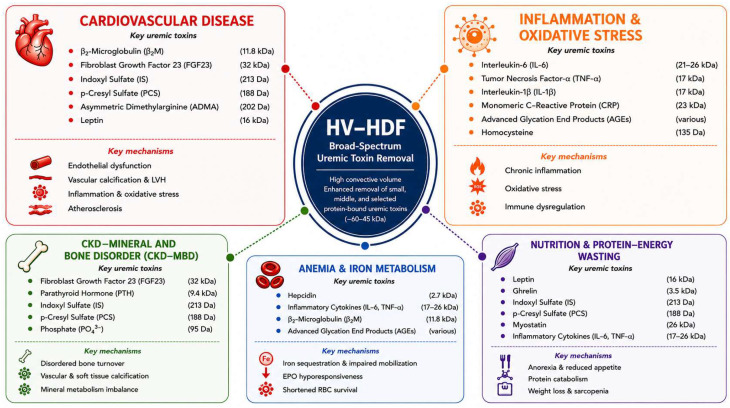
Uremic toxins removed by HVHDF and their clinical impact on ESKD complications.

Uremic toxins contribute to multiple complications of end-stage kidney disease through interconnected pathophysiologic pathways. Figure 3 summarizes representative toxins that are more effectively removed by HVHDF and their associations with major clinical domains, including inflammation and oxidative stress, CKD–mineral and bone disorder, cardiovascular disease, anemia, nutritional abnormalities, physical function, and quality of life. The following sections review the role of these toxins and the potential impact of HVHDF-enhanced removal on each domain.

## 6. Inflammation and Oxidative Stress

Chronic systemic inflammation and oxidative stress are hallmarks of the uremic milieu and play a central role in the development and progression of other common complications in ESKD patients, including cardiovascular disease, endothelial dysfunction, protein-energy wasting, immune dysregulation, and excess mortality in ESKD [119]. Compared with conventional HD, HDF has consistently been associated with lower inflammatory and oxidative stress burden [120]. 

These benefits likely result from the combined effects of improved membrane biocompatibility, the use of ultrapure dialysis fluid, and enhanced removal of middle- and large-MW solutes. By reducing circulating levels of pro-inflammatory mediators and other biologically active uremic toxins, HDF may attenuate the chronic systemic inflammation characteristic of ESKD in adults and children [6,37,40,44,57,58,120,121,122,123,124].

Several inflammation-related middle molecules that are more effectively reduced during HVHDF have been implicated in endothelial injury, oxidative stress, vascular dysfunction, and immune activation. These include interleukin-1 (IL-1), interleukin-6 (IL-6), tumor necrosis factor-α (TNF-α), complement activation products, free light chains, and other inflammatory mediators [6,37,40,44,57,58,59,60,61,62,63,64,66,67,68,69,70,71,75,120,121,122,123,124,125,126].

Advanced glycation end products (AGEs; >10 kDa) are strongly associated with oxidative stress, vascular injury, endothelial dysfunction, and cardiovascular complications. Plasma AGE concentrations have been reported to decrease in both diabetic and non-diabetic patients with ESKD treated with HVHDF [44,45].

Complement activation products, including C3a, C5a, and adipsin (~8–24 kDa), are also implicated in chronic inflammation and immune dysregulation in dialysis patients [37].

Interleukin-1α (17.5 kDa) contributes to endothelial dysfunction, accelerated atherosclerosis, erythropoietin resistance, and protein-energy wasting in dialysis patients [57,126].

Interleukin-18 (20 kDa) has similarly been associated with oxidative stress, endothelial injury, and immune dysregulation [58,59,60,61,62,63,64].

IL-6 (24.5 kDa) represents one of the most clinically relevant inflammatory mediators in dialysis populations. Elevated IL-6 concentrations are strongly associated with vascular inflammation, atherosclerotic progression, hospitalization, and cardiovascular mortality [66,67,68].

Free kappa (25 kDa) and lambda (25 kDa) immunoglobulin light chains accumulate progressively in kidney failure and are associated with chronic inflammation, vascular calcification, and immune dysfunction [69,70,71]. Maduell et al. demonstrated that reduction ratios of free light chains increased progressively across dialysis modalities, with the highest removal achieved during post-dilution HDF [24].

TNF-α (26 kDa) is a mediator of vascular inflammation and endothelial injury [127,128]. Elevated circulating TNF-α concentrations have been associated with coronary artery disease progression and adverse cardiovascular outcomes. TNF-α also promotes hepatic synthesis of fibrinogen and factor VIII, thereby contributing to the prothrombotic state observed in ESKD [58].

Alpha-1-acid glycoprotein (43 kDa), an acute-phase reactant synthesized predominantly by the liver, has also been associated with inflammation, malnutrition, and hypoalbuminemia in dialysis patients [70,75].

Native pentameric C-reactive protein (CRP) is a 115 kDa acute-phase protein composed of five identical 23 kDa subunits. It is synthesized by the liver in response to inflammation, primarily through IL-6 signaling. Because of its large molecular size, CRP is not directly removed by dialysis, including HDF. Consequently, lower CRP concentrations observed with HDF are generally considered to reflect a reduction in systemic inflammatory burden rather than direct extracorporeal clearance. A systematic review and meta-analysis of 24 randomized controlled trials found that HDF was associated with lower pre-dialysis CRP concentrations than conventional HD [27]. Across 12 randomized controlled trials including 3508 patients with ESKD, HDF reduced CRP by a weighted mean difference of −0.94 mg/L (95% CI −1.53 to −0.35; *p* < 0.01), although substantial heterogeneity was present (I^2^ = 79.8%) [27]. The effect was consistent across study designs, dialyzer membrane types, and substitution modes, and greater reductions were observed in studies with follow-up of 6 months or less, and meta-regression demonstrated an independent association between higher convective volumes and larger decreases in CRP [27].

P-cresyl sulfate (187 Da) and indoxyl sulfate (212 Da) are protein-bound uremic toxins (PBUTs) strongly implicated in chronic inflammation, oxidative stress, endothelial dysfunction, vascular calcification, and increased cardiovascular and all-cause mortality in ESKD patients. These solutes exert multiple adverse biologic effects, including activation of oxidative and inflammatory pathways, endothelial injury, vascular smooth muscle cell dysfunction, and progression of atherosclerotic and pro-calcific processes [30]. Compared with conventional high-flux HD, HVHDF has demonstrated enhanced removal of indoxyl sulfate and p-cresyl sulfate [28,29,30]. In a post hoc analysis of the HDFIT trial, patients treated with HVHDF who achieved convection volumes >27.5 L/session showed progressive reductions in predialysis indoxyl sulfate concentrations over six months, along with significantly lower p-cresyl sulfate levels compared with high-flux HD [30]. These findings support the concept that high convective transport may improve the extracorporeal handling of selected PBUTs despite their substantial protein binding.

The improved inflammatory profile associated with HVHDF may partly reflect the combined use of ultrapure dialysis fluid and sterile substitution fluid, both produced through validated ultrapurification systems incorporating bacteria- and endotoxin-retentive filters [129,130,131,132,133,134,135]. Post-dilution HVHDF operates under sustained net ultrafiltration conditions that largely prevent transmembrane pressure reversal and significant backfiltration. Consequently, convective transport is achieved through the controlled infusion of sterile, non-pyrogenic substitution fluid rather than by dialysate backfiltration. This may reduce the clinical relevance of uncontrolled backfiltration and the potential backtransport of pyrogenic substances, endotoxins, bacterial fragments, and other microbiological contaminants across the dialysis membrane, thereby attenuating chronic microinflammation, endothelial activation, cytokine signaling, oxidative stress, and endothelial dysfunction [135,136,137,138,139].

## 7. CKD–Mineral and Bone Disorder

Several uremic solutes implicated in CKD–mineral and bone disorder (CKD-MBD), including phosphate, parathyroid hormone fragments, and fibroblast growth factor 23 (FGF23), demonstrate enhanced extracorporeal removal during HVHDF. These compounds contribute to abnormal bone turnover, vascular calcification, endothelial dysfunction, and left ventricular hypertrophy.

Phosphate (95 Da) remains one of the most difficult uremic solutes to control in dialysis patients despite its relatively small molecular size. The in vitro mass transfer area coefficient (K0A) of phosphate is estimated to be only 40–60% that of urea [140]. During dialysis, phosphate removal occurs primarily from the plasma compartment, whereas intracellular phosphate mobilization remains relatively slow. Consequently, phosphate kinetics differ substantially from those of urea, and treatment duration becomes a major determinant of total phosphate removal [140]. Compared with conventional HD, HDF increases phosphate removal through additional convective transport throughout the dialysis session [27,141]. Kinetic modeling predicts that HVHDF may increase phosphate clearance by approximately 15–20% compared with conventional HD [27,44,141,142,143,144,145,146,147]. This enhanced removal may reduce phosphate-binder requirements in some patients [141]. However, greater extracorporeal phosphate clearance does not necessarily translate into lower predialysis serum phosphate concentrations [141]. Several non-dialytic factors strongly influence phosphate balance, including dietary phosphorus intake, intestinal absorption, phosphate-binder use, nutritional status, and residual kidney clearance of phosphate [38,141,146]. When these determinants are maintained constant, kinetic models predict an approximately 0.5 mg/dL reduction in predialysis phosphate concentrations with HVHDF compared with HD [141]. However, relatively small variations in phosphate intake, phosphate-binder prescription, or residual kidney function may offset this effect [141]. For example, an approximately 8% increase in phosphate intake, a reduction in the phosphate-binder equivalent dose by 1.5 g/day, or a decrease in residual kidney phosphate clearance by 0.8 mL/min may abolish the expected phosphate reduction associated with HDF [141]. These observations likely explain why several clinical studies reported similar predialysis phosphate concentrations among patients treated with HDF and conventional HD, despite higher phosphate clearance with HDF. Phosphate also demonstrates characteristic rebound kinetics following dialysis [38,148,149,150]. During treatment, serum phosphate concentrations progressively reach a plateau phase beyond which further reductions become limited. Following completion of dialysis, redistribution of intracellular phosphate into the extracellular compartment leads to post-dialytic rebound, a phenomenon observed in both HD and HDF [150]. These kinetic considerations are important when interpreting the phosphate-lowering effects of HDF in routine clinical practice.

β2-microglobulin (β2M, 11.8 kDa) is the best characterized middle molecule in dialysis patients. In ESKD, β2M accumulates progressively and forms fibrillary amyloid deposits within bones, periarticular tissues, vessel walls, and internal organs, particularly the heart. Elevated β2M concentrations have been strongly associated with dialysis-related amyloidosis, carpal tunnel syndrome, osteoarticular complications, cardiovascular disease, infectious complications, and increased mortality risk [26,46,47,48,49,50,51,52,53]. Elevated β2M concentrations alone do not fully explain the development of dialysis-related amyloidosis, as demonstrated by experimental studies [38,151]. The pathogenesis of dialysis-related amyloidosis is multifactorial and involves the accumulation of molecules that alter β2M conformation and promote the stabilization of amyloid fibrils. This process is further facilitated by the chronic inflammatory and oxidative stress milieu characteristic of uremia [38,152,153]. Accordingly, the potential advantage of HDF over conventional high-flux HD may extend beyond enhanced β2M removal and include attenuation of the persistent inflammatory and oxidant state that contributes to amyloidogenesis. Clinical observations have also suggested that these combined effects may help to improve osteoarticular manifestations following transition from HD to high-volume HDF.

Enhanced removal of middle- and larger-MW uremic toxins has been associated with reduction in joint pain and improvement in extremity mobility in patients with dialysis-related amyloidosis and related musculoskeletal complications [38]. Compared with conventional HD, HDF demonstrates substantially greater β2M removal and produces significant reductions in circulating β2M concentrations over time [48,49,50,51,52,53]. In a post hoc analysis of the HEMO study, predialysis β2M concentrations were independently associated with mortality risk, with risk increasing substantially above approximately 27 mg/L and rising by up to 60% at concentrations of 50 mg/L [26,46,47]. The MPO study subsequently demonstrated improved survival among diabetic and hypoalbuminemic patients treated with high-flux HD, further supporting the clinical importance of middle-molecule removal [26,154]. These findings contributed to the incorporation of β2M targets into European and Japanese dialysis guidelines [155,156]. The Japanese Society for Dialysis Therapy recommended predialysis β2M concentrations <25 mg/L for HDF and <27 mg/L for high-flux HD [156].

Reported β2M reduction ratios vary substantially according to dialysis modality, ranging from approximately 50–60% with high-flux HD to approximately 70% with MCO membranes and 80–85% with HVHDF [100,108,157]. HVHDF may therefore be particularly beneficial in patients with β2M concentrations ≥ 27 mg/L or in patients with symptomatic dialysis-related amyloidosis, including arthropathy, bone cysts, carpal tunnel syndrome, autonomic dysfunction, and systemic involvement [46,51].

Ward et al. demonstrated that highly efficient HDF conditions achieved β2M clearances of approximately 73 mL/min, corresponding to nearly 200 mg removed during a single 4 h session [158]. β2M kinetic analyses consistently show that HDF provides approximately 30% greater β2M removal than conventional high-flux HD [26,158,159,160,161]. Because β2M clearance is directly related to convective volume, HVHDF currently appears to achieve the highest extracorporeal β2M clearance among conventional intermittent dialysis modalities [49,162,163]. A large meta-analysis published in 2018 evaluated determinants of β2M removal across 69 studies involving 1879 patients and 6771 clearance measurements [51]. Convective therapies significantly outperformed conventional high-flux HD, achieving mean β2M clearances of approximately 87 mL/min compared with 49 mL/min for high-flux HD [51]. Membrane composition (high-flux dialyzers composed of polyarylethersulfone exhibited superior β2M clearance in high-flux HD, whereas polysulfone was associated with better performance in HDF), blood flow, and substitution volume emerged as major determinants of β2M removal [51]. The study also demonstrated an important contribution of membrane adsorption to the elimination of β2M. Dialysate-side β2M clearances were substantially lower than whole-blood clearances, suggesting that adsorption contributes significantly to total β2M removal beyond purely diffusive and convective mechanisms [51].

Wathanavasin et al. found that, across 16 randomized controlled trials including more than 3000 patients with ESKD, HDF was consistently associated with lower serum β2M concentrations than conventional HD, despite substantial between-study heterogeneity [27]. Greater reductions were observed with higher convective volumes [27].

Predialysis β2M concentrations may not differ substantially between HDF and high-flux HD immediately after conversion to HDF, as several weeks (typically 4–6 weeks) are required to establish a new steady-state concentration [164]. Once equilibrium is reached, predialysis ß2M levels are strongly influenced by the total weekly convective volume delivered, as predicted by kinetic modeling [165], and confirmed in real-world study [95]. The principal limitation to β2M removal during post-dilution HDF is not the clearance capacity of the hemodiafilter itself, but rather resistance to intercompartmental mass transfer and tissue to plasma mobilization within the patient [166]. During dialysis, β2M is rapidly removed from well-perfused compartments, whereas mobilization from remote tissue stores remains relatively slow. This compartmentalization effect limits total extracorporeal extraction even during HVHDF [166]. Following dialysis, redistribution of β2M from peripheral compartments into the circulation results in post-dialytic rebound [166]. These observations underscore the intrinsic difficulty of removing tissue-sequestered middle molecules despite the high efficiency of extracorporeal therapies.

Parathyroid hormone fragments (~9 kDa) and FGF23 (26–32 kDa) are implicated in CKD bone metabolism alterations. Elevated levels of these molecules contribute to CKD-related mineral and bone disorders (CKD-MBD), leading to complications such as renal osteodystrophy and vascular calcification. Enhanced removal of these molecules during HVHDF may contribute to attenuation of CKD-MBD-related biochemical abnormalities and its associated complications and may attenuate the need for calcimimetics in selected patients [38,39,40,41,42,43].

## 8. Cardiovascular Health and Intradialytic Hemodynamic Tolerance

Cardiovascular disease remains the leading cause of morbidity and mortality in dialysis patients. Although traditional cardiovascular risk factors contribute substantially to this burden, the retention of middle molecules and other biologically active uremic solutes also plays an important role in myocardial remodeling, vascular calcification, endothelial dysfunction, and progressive cardiovascular injury. As discussed in the previous section, chronic inflammation and oxidative stress are central mechanisms linking retained uremic toxins with cardiovascular disease.

The cardiovascular benefits associated with HVHDF are unlikely to depend on the removal of a single uremic toxin. Instead, they are more likely to reflect the combined reduction of multiple biologically active solutes together with the physiological effects of convection-based therapy, including improved sodium balance, enhanced plasma refilling, better thermal balance, the use of ultrapure dialysate and sterile substitution fluid, attenuation of chronic inflammatory activation, improved endothelial function, and greater intradialytic hemodynamic stability [167]. Progressive retention of middle molecules implicated in inflammation, oxidative stress, endothelial dysfunction, myocardial remodeling, and vascular injury contributes substantially to cardiovascular risk in ESKD.

Elevated β2M concentrations are associated not only with dialysis-related amyloidosis, but also with increased cardiovascular morbidity and mortality in dialysis patients [1,26,46]. Enhanced β2M clearance during HDF likely reflects improved global removal of middle molecules with potential cardiovascular toxicity [26].

Inflammatory cytokines, including IL-1β, TNF-α, and IL-6, participate in endothelial activation, nitric oxide-mediated vasodilation, vascular stiffness, myocardial dysfunction, and intradialytic hemodynamic instability [32,168,169,170,171,172]. Chronic inflammatory activation has additionally been associated with impaired vascular responsiveness and intradialytic hypotension [168,169].

FGF23 represents another middle molecule of major cardiovascular relevance. Elevated FGF23 concentrations have been strongly associated with left ventricular hypertrophy, myocardial fibrosis, arrhythmogenesis, and mortality in dialysis populations [72,173,174]. Several studies suggest improved FGF23 removal during HDF compared with conventional HD [43], although the clinical significance of this finding remains uncertain.

Other uremic solutes, including free immunoglobulin light chains, β-trace protein, YKL-40, indoxyl sulfate, and p-cresyl sulfate, have also been associated with endothelial dysfunction, vascular injury, inflammation, and adverse cardiovascular outcomes. β-trace protein (23–29 KDa), also known as prostaglandin D synthase, is involved in prostaglandin metabolism and transport processes. Elevated concentrations have been associated with adverse cardiovascular outcomes in CKD and ESKD [65]. HDF has been shown to reduce circulating β-trace protein concentrations more effectively than conventional HD [70]. YKL-40 (40 kDa) has been linked to vascular inflammation, endothelial dysfunction, and atherosclerotic disease [73,74]. PBUTs such as indoxyl sulfate and p-cresyl sulfate also contribute to endothelial dysfunction, oxidative stress, vascular calcification, and cardiovascular toxicity [28,29,30]. A recent review has highlighted the role of gut-derived uremic toxins, including p-cresyl sulfate, indoxyl sulfate, indole-3-acetic acid, trimethylamine N-oxide, and phenylacetylglutamine, in the pathogenesis of cardiovascular disease in CKD. Their accumulation promotes oxidative stress, inflammation, endothelial dysfunction, and vascular injury and has been associated with an increased risk of cardiovascular disease [175]. However, evidence that lowering these toxins improves clinical outcomes remains limited [175].

In addition to enhanced middle-molecule removal, several complementary physiologic mechanisms may contribute to the superior hemodynamic tolerance frequently observed during HDF [96]. Enhanced convective transport modifies sodium handling via the Gibbs–Donnan effect, thereby favoring the preservation of plasma osmolality and improved vascular refilling from the interstitial compartment during ultrafiltration [176,177,178]. Sodium balance remains generally well preserved during post-dilution HDF despite large substitution volumes [11,179,180]. Improved endothelial stability associated with isotonic bicarbonate-buffered substitution fluid and high microbiological purity may also contribute [167,181,182]. Mild extracorporeal cooling during dialysis may additionally improve blood pressure stability [183,184].

The use of ultrapure dialysate and sterile substitution fluid may also indirectly improve cardiovascular tolerance by reducing chronic inflammatory stimulation and endothelial activation. Even low-grade endotoxin exposure during dialysis has been associated with persistent monocyte activation, increased production of inflammatory cytokines, including TNF-α and IL-6, endothelial dysfunction, and accelerated atherosclerosis [129,130,131,136,137].

Chronic inflammatory activation may also impair vascular reactivity and contribute to intradialytic hemodynamic instability through nitric oxide-mediated vasodilation and autonomic dysfunction. A reduction in chronic inflammatory stimulation may therefore indirectly contribute to improved endothelial stability, vascular responsiveness, and intradialytic hemodynamic tolerance during HVHDF.

Randomized clinical trials, meta-analyses, and observational studies have reported better intradialytic hemodynamic tolerance with HVHDF than with conventional HD, including a lower incidence of intradialytic hypotension [96]. The relative contribution of the individual mechanisms responsible for these effects cannot be determined. However, enhanced removal of middle molecules, reduced inflammatory activation, improved sodium and fluid balance, preservation of endothelial function, use of ultrapure dialysate and sterile substitution fluid, and thermal balance probably all contribute. The cardiovascular effects of HVHDF should therefore not be attributed to the removal of a single uremic toxin, but rather to the combined effects of these mechanisms.

## 9. Anemia and Nutritional Abnormalities

Anemia, metabolic dysfunction, and protein-energy wasting are highly prevalent complications in patients with ESKD and are strongly associated with inflammation, oxidative stress, cardiovascular disease, hospitalization, impaired quality of life, and mortality. Accumulation of middle- and large-MW uremic toxins contributes to erythropoietin resistance, impaired iron metabolism, anorexia, muscle wasting, endocrine dysfunction, insulin resistance, and chronic catabolism.

HDF has been associated with improved anemia control and lower erythropoiesis-stimulating agent (ESA) requirements compared with conventional HD [33,124,185,186,187,188,189]. Across two randomized controlled trials including 906 patients with ESKD, Wathanavasin et al. showed that HDF was associated with a significant reduction in weekly erythropoietin dose (weighted mean difference −587.8 units/week; 95% CI −917.1 to −258.5; *p* < 0.01) [27]. These effects are likely multifactorial and may involve enhanced removal of inflammatory and erythropoiesis-inhibiting middle molecules, attenuation of oxidative stress, improved iron mobilization, increased phosphate removal, and prolonged red blood cell survival. Although several studies support improved ESA responsiveness during HDF, large, randomized trials specifically designed to evaluate anemia-related outcomes remain limited.

Among the molecules implicated in anemia resistance, hepcidin (2.8 kDa) plays a central role in iron dysregulation. Hepcidin inhibits intestinal iron absorption and macrophage iron release, thereby contributing to functional iron deficiency and ESA hypo-responsiveness. Several studies have shown lower circulating hepcidin concentrations and improved iron utilization during online HDF [31,32,33,34,35,36].

Inflammatory cytokines, including IL-6, TNF-α, and IL-1β, additionally contribute to anemia by suppressing erythroid progenitor proliferation, impairing erythropoietin signaling, and stimulating hepcidin synthesis [31,32]. Other incompletely characterized erythropoiesis-inhibiting middle molecules may also accumulate during kidney failure [190]. Enhanced convective removal of these substances may contribute to improved hematologic response during HVHDF, although the precise identity and relative contribution of these toxins remain only partially understood.

Improved phosphate removal during HVHDF may also indirectly contribute to better anemia control by attenuating secondary hyperparathyroidism and bone marrow suppression. Furthermore, HDF has been associated with prolonged red blood cell survival and reduced hemolysis-related anemia in small mechanistic studies [191]. However, additional adequately powered randomized studies are required to define the long-term clinical significance of these observations.

Protein-energy wasting and malnutrition-inflammation complex syndrome are common in dialysis patients and are associated with sarcopenia, frailty, impaired immune function, poor quality of life, and reduced survival [192,193]. Chronic inflammation and accumulation of anorexigenic and catabolic middle molecules play central roles in this process. Several studies have associated HVHDF with improved appetite, higher protein intake, preservation of lean tissue mass, and improved nutritional markers compared with conventional HD [192,194,195]. Among the most relevant molecules is leptin (16 kDa), an anorexigenic cytokine-like hormone that accumulates in ESKD and contributes to appetite suppression, inflammation, increased energy expenditure, and protein-energy wasting [54,55,56]. Enhanced leptin removal during HDF may contribute to improved appetite and nutritional status, although the precise clinical relevance of leptin clearance remains incompletely established.

Inflammatory cytokines, including IL-6, TNF-α, and IL-1β, additionally contribute to muscle catabolism, anorexia, hypoalbuminemia, and cachexia. Improved hemodynamic stability, lower inflammatory burden, attenuation of oxidative stress, and better correction of metabolic acidosis may indirectly contribute to preservation of lean tissue mass and nutritional status during HDF [196,197,198].

## 10. Clinical Outcomes: From Uremic Toxin Removal to Patient Benefit

Numerous observational studies have shown that elevated circulating concentrations of middle molecules and protein-bound uremic toxins are associated with cardiovascular disease, chronic inflammation, hospitalization, and mortality in patients receiving dialysis. However, these associations should not be interpreted as evidence that lowering an individual uremic toxin improves clinical outcomes.

Although β2M is the best-established marker of middle-molecule retention and is consistently associated with adverse clinical outcomes, no individual uremic toxin has been validated as a surrogate endpoint for morbidity or mortality in patients receiving dialysis. Consequently, improvements in toxin clearance should be regarded as biological markers of treatment efficacy rather than direct measures of clinical benefit. For this reason, the clinical effects of HDF should be assessed based on randomized controlled trials evaluating patient-centered outcomes rather than inferred from changes in biochemical markers alone. Early randomized trials consistently demonstrated greater middle-molecule removal with HDF than with conventional HD.

The CONVINCE trial demonstrated a significant reduction in all-cause mortality with high-dose HDF compared with high-flux HD [12]. Subsequently, an individual participant data meta-analysis published in The Lancet, including 4153 patients from five randomized controlled trials, confirmed significant reductions in both all-cause and cardiovascular mortality with HDF [14]. Importantly, the meta-analysis found no evidence of heterogeneity in treatment effect across the major predefined patient subgroups and demonstrated a dose–response relationship between delivered convection volume and survival, with progressively greater clinical benefit observed at higher delivered convection volumes. These findings further support the importance of delivering an adequate convective dose to maximize the clinical benefits of HDF.

The randomized trials enrolled heterogeneous maintenance dialysis populations representative of contemporary clinical practice, including predominantly older patients with a high prevalence of diabetes, cardiovascular disease, and other comorbidities, all of which are major determinants of prognosis in dialysis patients [8,9,10,11,12]. Across these studies, major clinical outcomes included all-cause mortality, cardiovascular mortality, and patient-reported quality of life, thereby extending the evaluation of HDF beyond biochemical endpoints alone. Although subgroup analyses should be interpreted with appropriate caution, the available randomized evidence does not suggest that the clinical effects of HDF are confined to specific patient populations.

The available evidence does not support the concept that the clinical benefits of HDF result from enhanced removal of any individual uremic toxin. Rather, they are more likely to reflect the combined effects of improved removal of a broad spectrum of retained solutes together with several physiological effects of convection-based therapy, including reduced chronic inflammatory activation, improved intradialytic hemodynamic stability, optimized sodium and fluid balance, and the use of sterile substitution fluid. The survival benefit observed with HDF should not be interpreted as evidence that enhanced removal of β2M, or any other individual uremic toxin, is the sole mechanism responsible for the observed clinical benefit.

The clinical rationale for HDF is therefore supported by evidence from randomized controlled trials and individual participant data meta-analyses demonstrating improvements in hard clinical outcomes rather than by improvements in individual biochemical markers alone.

## 11. Conclusions

Contemporary understanding of uremic toxicity extends far beyond small-solute kinetics and increasingly emphasizes the biologic importance of middle molecules and other retained uremic solutes. Conventional diffusion-based dialysis incompletely removes many of the compounds implicated in inflammation, oxidative stress, cardiovascular injury, immune dysfunction, anemia, mineral-bone abnormalities, and protein-energy wasting.

HVHDF combines efficient diffusive clearance of small solutes with enhanced convective removal of small and middle molecules across a broad MW spectrum up to albumin. Its benefits are further supported by the use of highly biocompatible membranes, a well-engineered hemodialyzer, ultrapure dialysis fluid, and sterile, non-pyrogenic substitution fluid. Growing evidence indicates that high convective volumes improve the clearance of several biologically relevant middle molecules, including β2-microglobulin, inflammatory cytokines, free light chains, hepcidin, leptin, β-trace protein, FGF23, and other larger uremic toxins. These observations are consistent with the results of a systematic review and meta-analysis, which reported greater removal of several uremic toxins and inflammatory markers, together with lower erythropoiesis-stimulating agent requirements during HDF compared with conventional HD [27].

Importantly, dialysis efficiency depends not only on the modality and convective volume but also on the treatment schedule. Increasing total weekly treatment time remains a key determinant of solute removal, hemodynamic tolerance, volume control, and overall clinical outcomes. Longer and more frequent treatment schedules enhance the clearance of slowly equilibrating solutes, improve cardiovascular stability, and better mimic the continuous nature of native kidney function. Preservation of residual kidney function further complements these benefits by facilitating the removal of middle molecules and protein-bound uremic toxins that are less efficiently cleared by extracorporeal therapies.

Although several mechanistic pathways remain incompletely understood, the biologic rationale supporting HVHDF continues to strengthen. Enhanced middle-molecule removal, attenuation of chronic inflammatory activation and oxidative stress, improved endothelial function, and cardiovascular stability, together with the potential for better long-term clinical outcomes, provide a compelling framework for the broader adoption of optimized HVHDF. Successful implementation requires an optimized dialysis prescription, including adequate treatment time, blood flow, and delivered convection volume. It also depends on appropriate dialysis infrastructure, reliable water treatment systems, adequate vascular access capable of supporting high blood flow rates, and trained healthcare personnel. Differences in healthcare resources, reimbursement policies, and local infrastructure may limit implementation in some settings and should be considered when translating the available evidence into routine clinical practice.

Future advances in dialysis adequacy assessment should therefore extend beyond traditional small-solute metrics to incorporate measures that better reflect the removal of clinically relevant middle- and large-molecular-weight toxins.

## Figures and Tables

**Table 1:** Main uremic toxins removed by hemodiafiltration (HDF).

Uremic Toxin	MW	Main Biological/Clinical Effects in ESKD	Relative Removal by HDF
Phosphate	95 Da	CKD-MBD, SHPT, VC	Improved vs. HD
PB p-Cresyl sulfate [28,29,30]	187 Da	Chronic INF, vascular toxicity, CV mortality	Improved with HVHDF
PB Indoxyl sulfate [28,29,30]	212 Da	Oxidative stress, VC, endothelial dysfunction	Improved with HVHDF
Hepcidin [31,32,33,34,35,36]	2.8 kDa	ESA resistance, impaired iron mobilization	Good
Complement products [37]	~8–24 kDa	Chronic INF, immune dysregulation	Good
PTH fragments [38,39,40,41,42,43]	9.2 kDa	CKD-MBD, bone disease, VC	Good
AGEs [44,45]	>10 kDa	Endothelial dysfunction, vascular injury, INF	Moderate to good
β2-microglobgulin [26,46,47,48,49,50,51,52,53]	11.8 kDa	DRA, CV disease, mortality	Excellent
Leptin [54,55,56]	16 kDa	Appetite suppression, PEW, INF	Good
Interleukin-1α [57]	17.5 kDa	Endothelial dysfunction, ESA resistance	Moderate
Interleukin-18 [58,59,60,61,62,63,64]	20 kDa	Oxidative stress, immune dysregulation	Moderate
β-trace protein [65]	~23–29 kDa	CV risk, endothelial dysfunction	Good
Interleukin-6 [66,67,68]	24.5 kDa	Systemic INF, CV mortality	Moderate
Free lambda light chains [69,70,71]	25 kDa	Chronic INF, endothelial dysfunction	Good with HVHDF
Free kappa light chains [69,70,71]	25 kDa	INF, VC	Good
TNF-α [58]	26 kDa	Vascular INF, thrombosis, endothelial injury	Moderate
FGF 23 [39,72]	26–32 kDa	LV hypertrophy, myocardial fibrosis, VC	Good
YKL-40 [73,74]	40 kDa	Vascular INF, atherosclerosis	Moderate to good
α-1-acid glycoprotein [70,75]	43 kDa	INF, malnutrition, hypoalbuminemia	Moderate

Legend. ESKD: End-Stage Kidney Disease; CKD-MBD: Chronic Kidney Disease–Mineral and Bone Disorder; SHPT: Secondary Hyperparathyroidism; VC: Vascular Calcification; ESA: Erythropoiesis-Stimulating Agent; CV: Cardiovascular; LV: Left Ventricular; MW: Molecular Weight; PB: Protein-Bound; Complement products: C3a, C5a, adipsin; PTH: Parathyroid hormone; AGEs: Advanced Glycation End products; DRA: Dialysis-Related Amyloidosis PEW: protein-energy wasting; FGF 23: Fibroblast Growth Factor 23; YKL-40: Chitinase-3-like protein 1; HVHDF: High-Volume HDF.

**Table 2:** Equations for solute clearance with hemodiafiltration.

Parameter	Formula		Description
1. Plasma flow (Q_p_)	Q_p_ = Q_b_ × (1 − hematocrit/100)	(1)	Plasma flow is derived from blood flow and hematocrit
2. Plasma CL (K_p_)	K_p_ = (C_art_ × Q_p.art_ − C_ven_ × Q_p.ven_)/C_art_	(2)	Total clearance calculated from plasma concentrations and flows across the dialyzer
3. Dialysate CL (K_d_)	K_d_ = C_dial out_ × Q_d,out_/C_art_	(3)	Total clearance calculated from the dialysate side
4. Theoretical Diffusive CL (K_diff_)	$K_{diff}=Q_{b,art}\frac{1-e^{\frac{K_{0}A}{Q_{b,art}}\left(1-\frac{Q_{b,art}}{Q_{d,in}}\right)}}{\frac{Q_{b,art}}{Q_{d,in}}-e^{\frac{K_{0}A}{Q_{b,art}}\left(1-\frac{Q_{b,art}}{Q_{d,in}}\right)}}$	(4)	Clearance in HDF is modeled in 2 consecutive steps: 1. step: Diffusion. Diffusive solute clearance from the intrinsic characteristics of the dialyzer and flow rates
5. Theoretical Convective CL (K_conv_)	K_conv_ = [(Q_b,art_ − K_diff_)/Q_b,art_] × Q_f_ × S	(5)	2. step: Convection. Convective solute clearance from the intrinsic sieving characteristics of the dialyzer and flow rates

Legend. CL: Clearance; CQ_b,art_ and Q_b,ven_ = arterial and venous blood (water) flow rate (Q_b,art_ = Q_b,ven_ + Q_uf_); Q_p,art_ and Q_p,ven_ = arterial and venous plasma flow rate (Q_p,art_ = Q_p,ven_ + Q_uf_); Q_d,in_ and Q_d,out_ = dialysate flow rate at dialyzer inlet and outlet (Q_d,out_ = Q_d,in_ + Q_uf_); Q_f_ = convection flow rate (Q_f_ = Q_sub_ + Q_uf_); Q_sub_ = substitution flow; Q_uf_ = weight loss flow; K_p_ = plasma clearance; C_art_ =arterial (pre-dialyzer) blood solute concentration; C_ven_ = venous (post-dialyzer) blood solute concentration; C_dial out_ = solute concentration at dialyzer outlet; K_0_A = solute-specific membrane mass transfer-area coefficient; S = solute specific sieving coefficient of membrane.

## Data Availability

No new data were created or analyzed in this study.
